# Epigenetics changes caused by the fusion of human embryonic stem cell and ovarian cancer cells

**DOI:** 10.1042/BSR20160104

**Published:** 2016-09-16

**Authors:** Ke He, Hu Qu, Li-Nan Xu, Jun Gao, Fu-Yi Cheng, Peng Xiang, Can-Quan Zhou

**Affiliations:** * Department of Obstetrics and Gynecology, the First Affiliated Hospital of Sun Yat-Sen University, GuangZhou 510080, China; † Department of Urology, the Sixth Affiliated Hospital of Sun Yat-Sen University, GuangZhou 510655, China; ‡ Reproductive Medicine Research Center, the Sixth Affiliated Hospital of Sun Yat-Sen University, GuangZhou 510655, China; § Reproductive Medical Center, the First Affiliated Hospital of Sun Yat-Sen University, GuangZhou 510080, China; ║ Center for Stem Cell Biology and Tissue Engineering, Sun Yat-Sen University, Guangzhou 510080, China

**Keywords:** epigenetics, fusion, human embryonic stem cell, ovarian cancer cell, p53, PTEN

## Abstract

To observe the effect of gene expression and tumorigenicity in hybrid cells of human embryonic stem cells (hESCs) and ovarian cancer cells *in vitro* and *in vivo* using a mouse model, and to determine its feasibility in reprogramming tumour cells growth and apoptosis, for a potential exploration of the role of hESCs and tumour cells fusion in the management of ovarian cancer. Stable transgenic hESCs (H1) and ovarian cancer cell line OVCAR-3 were established before fusion, and cell fusion system was established to analyse the related indicators. PTEN expression in HO-H1 cells was higher than those in the parental stem cells and lower than those in parental tumour cells; the growth of OV-H1 (RFP+GFP) hybrid cells with double fluorescence expressions were obviously slower than that of human embryonic stem cells and OVCAR-3 ovarian cancer cells. The apoptosis signal of the OV-H1 hybrid cells was significantly higher than that of the hESCs and OVCAR-3 ovarian cancer cells. *In vivo* results showed that compared with 7 days, 28 days and 35 days after inoculation of OV-H1 hybrid cells; also, apoptotic cell detection indicated that much stronger apoptotic signal was found in OV-H1 hybrid cells inoculated mouse. The hESCs can inhibit the growth of OVCAR-3 cells *in vitro* by suppressing p53 and PTEN expression to suppress the growth of tumour that may be achieved by inducing apoptosis of OVCAR-3 cells. The change of epigenetics after fusion of ovarian cancer cells and hESCs may become a novel direction for treatment of ovarian cancer.

## INTRODUCTION

Ovarian cancer, featured by rapid aggravation and insidious onset, is the most lethal gynaecologic malignancy, ranking as the fifth most frequent cause of death in females with cancers, which inevitably has a severe impact on public health [1,2]. According to statistics, more than 22000 new cases were diagnosed and approximately 14000 died of the disease in the United State population [3]. Due to a lacking of specific symptoms and effective early screening strategies, over 70% of the diagnoses are confirmed at advanced stages, and the average relative 5-year survival rates are extremely low [4,5]. For example, among 100 women who have suffered ovarian cancer, approximately 30 women will have a recurrence and die of the disease, and a recent research from the UK showed a 5-year survival rate of only close to half percentage [6,7]. The aetiology of ovarian cancer is multifactorial which can be both environmental and genetical, such as family history, genetic mutations, reproductive history, lifestyle factors (smoking or obesity), excessive gonadotropin secretion, as well as oestrogen and progesterone imbalance [8–11].

There is adequate evidence on the mechanism exploration of the development of ovarian cancer, but definitive explanation is still warranted. Recently, with the development of cancer stem cell research, it is found that tumour cells have many similarities to the stem cells [12], the cancer stem cell hypothesis is therefore proposed and merits more rigorous tests. The hypothesis insisted that the development and recurrence of tumours is mainly attributed to the unlimited proliferation of cancer stem cells, and cancer stem cells may come from the mutation or fusion of stem cells. Targeted therapy has been a hotspot for tumour treatment [13,14], which changes the site-directed genetic characteristics of cancer cells according to the therapeutic goal to treat the diseases [15–17]. The most important method for reconstruction of tumour cell specificity is reprogramming [18–20]. At present co-culture of stem cell extracts and cell fusion are the most common reprogramming methods [21–25]. After fusion, the cells have the genetic materials from the two parental cells, which will have new genetic or biological characteristics [26,27]. In the present study, we aimed to observe the changes of gene expression and tumorigenicity in hybrid cells of human embryonic stem cells (hESCs) and ovarian cancer cells *in vitro* and *in vivo*, and to determine if the tumour cells can be reprogrammed successfully, for a potential exploration of the role of hESCs and tumour cells fusion in the management of ovarian cancer.

## MATERIALS AND METHODS

### Ethics statement

Procedures involving animals and their care were conducted in conformity with NIH guidelines (NIH Pub. No. 85-23, revised 1996) and was approved by Animal Care and Use Committee of the First Affiliated Hospital of Sun Yat-Sen University.

### Preparation of mouse embryonic fibroblast (MEF) feeder layer

#### MEF feeder layer in primary culture

Pregnant Kunming mice (13.5 days, weighing 30±5 g) provided by Animal Center of the Sun Yat-Sen University were bred at this centre and maintained under standard laboratory conditions. The mice were killed by cervical dislocation, and then soaked with 75% alcohol for 5 min, placed in a sterile culture dish and removed the head, tail, limbs and organs, the remaining tissues were washed with PBS buffer (×2 times) containing penicillin (100 units/ml) and streptomycin (100 ug/ml). Tissues were then cut and digested with 0.25% trypsin at 37°C for 10 min. Terminated the digestion, 1000 rmp centrifugation was last for 5 min (two times) to clean the cells. After the centrifugation, these cells were cultured in Dulbecco's Modified Eagle's Medium (DMEM, Gibco BRL) containing 10% fetal calf serum at 37°C in 5% CO_2_ under humidity conditions.

#### MEF feeder layer in subculture

When the primary cultured cells were gradually converged, and the bottom of the bottle covered most of the surface, the first passage was performed. With 0.25% trypsin digestion cells added, cells were observed under microscope. When the contraction of the circular was observed under the microscope, the addition of the culture fluid in a ratio of 1:3 was used to terminate the digestion for the first generation. On behalf of the cell at the bottom of the bottle, some cells continued to subculture, some of the cells were collected and frozen. The cell density was 1×10^7^ ml and the frozen solution was 10% DMSO+40% fetal bovine serum+50% culture fluid at -80°C overnight in liquid nitrogen for long-term preservation.

#### Preparation of MEF feeder layer

The mouse embryonic fibroblast (MEF) feeder layer was prepared after continuously purifying through digestion and passage, and the 3–5 generation of MEF can be used as the ideal feeder layers. When the cells covered the bottom of the bottle, the cells were treated with culture liquid with 10 ug/ml MMC for 2 h at room temperature (37°C), and then washed with PBS for five times, followed by a 0.25% trypsin digestion and centrifugation to collect cells. Subsequently, the collected cells were transferred to the coated culture plates pre-treated with 0.1% galectin (2 ml).

### Cells line, experimental animals and reagents

Human epithelial ovarian cancer cell line OVCAR-3 and HO8910 were provided from the State Key Laboratory (SKL) of Oncology in South China of Sun Yat-Sen University and preserved in the Laboratory. Severe combined immunodeficiency (SCID) male mice, aged from 5 to 8 weeks, were provided by the Animal Center of the Sun Yat-Sen University. PEG1500 kit, DMEM culture medium, PRMI-1640 culture medium, DMEM/F-12 culture medium were obtained from Life Technology.

### Fusion of hESCs and ovarian cancer cells

Construction of plasmids co-expressing hESC (H1 was used as the short name in the experimental process) and OVCAR-3, HO8910 was performed. Gateway Technology was applied for the construction of pFinal/PGK-BSD-EF1α-hrGFP and pFinal/PGK-puro-EF1α-dTomato vector plasmid. AttB4-EF1-attB1R was amplified following PCR carried with attB1R and attB4, and then under the action of Clonase™ II Enzyme Mix, the BP site of the entry vector pDONRTMP4-P1R was generated, and the pUp-EF1 was cloned. Subsequently, attB1-hrGFP-attB2 was amplified carried with attB1 and attB2, and then under Clonase™ II Enzyme Mix action, BP action was occurred at the attP1 and attP2 site of the entry vector pDONRTM221, and the pUp-EF1 was cloned. PDown-tdTomato was also generated using the same way. Then, as for the construction of target vector, the blasticidin of pLenti6/block-iT-DEST was replaced by puromycin by using the enzyme digestion and ligation reaction, and the target vector pDestpuro was constructed. The pFinal/PGK-BSD-EF1α-hrGFP was also constructed by the same method after the construction of expression vectors with LR reaction. The 293FT cells with good growth state were selected, and the cells (5×10^6^) were inoculated in a 10-mm tissue culture dishes coated with 2% gelatin, virus packaging was performed when the cell density reached 90–95%.

The 20 ml viral supernatant was collected 48–72 h after retrovirus packaging, removal of cell debris was conducted with a 0.45 μm filter. Then, the lysate was cleared by ultracentrifugation at 50000 × ***g*** at 4°C for 1.5 h in an ultracentrifugation tube. When there was visible white spot of virus particles sedimentation in the tube at the bottom of the side wall, the supernatant was discarded and dissolved with 200 μl precooling PBS, and finally stored to -80°C for further usage. Virus RNA extraction by TIANamp viral RNA extraction kit (Tiangen) was performed in accordance with the manufacture's protocols. PCR reaction were then performed, followed by the inoculation of the well-growth hESCs into the prepared 12-well plate MEF layers for cell lines purification. HO8910 or OVCAR-3 ovarian cancer cells with good growth state were selected, and inoculated into 12-well plate. When the ovarian cancer cells were attached to the wall the next day, cells infected with the virus were selected when the density at 80–90%.

The established stable H1 hESCs, with blasticidin resistance and GFP fluorescence expression, were fused with ovarian cancer cells with puromycin resistance and RFP fluorescence expression, and before fusion the cells were digested by 0.25% pancreatin and counted. The ratio of H1 cells and ovarian cancer cells was 1:1. All the cells were preserved by slow freezing method for further usage. The hybrid cells OV-H1, HO-H1 fusion cell, as well as the parent cells, hESC and OVCAR-3, HO8910 ovarian cancer cells, were further observed for their growth and apoptosis situations.

### Detection of cell growth

Parental cells and the 12th generation hybrid cells were counted after digested by pancreatin. 1×10^6^ cells were inoculated in 6 cm culture dishes; each type of cells was inoculated in 21 dishes. Cells of three dishes were collected and counted to calculate the average value every 24 h for 7 days in total. The growth curve was constructed according to cell count result, and the doubling time of cell population was calculated according to the following formula: TD=*t*log 2/log(*N*/*N*_0_). *t* means the time from inoculation to detection, *N* means the total cell amount detected at *t* time point, *N*_0_ means the inoculated cell mount. The test was repeated three times for a much stable value collection.

### Cell apoptosis detection

Forty-eight hours after fusion, the cells were fixed with 4% paraformaldehyde, terminal dexynucleotidyl transferase (TdT)-mediated dUTP nick end labelling (TUNEL) assay was performed in accordance with the manufacture's guidelines (Nanjing KGI Biological Technology Development). A 4% goats serum (Beijing Zhongshan Jinqiao Biotechnology) was applied to seal the samples at room temperature for 10 min, 3% H_2_O_2_ was then added for 10 min to eliminate the endogenous risk of peroxidase. Following, the primary antibodies rabbit anti-human caspase-9 (1:1000) was added at 37°C for 1 h under humid conditions; after washed with buffer solution, mouse anti-rabbit IgG horse-radish peroxidase-labelled secondary antibody (1:1000) was added at 37°C for 30 min under humid conditions, followed by the other buffer solution washing. Both the primary and secondary antibodies were purchased from the Nanjing KGI Biological Technology Development. Finally, cells apoptosis imaging was visualized by 3,3′-diaminobenzidine tetrahydrochloride (DAB, obtained from Fuzhou Maixin Biotechnology Development) colour and observed under microscope. Experimental procedures were repeatedly observed three times.

### Real-time fluorescence quantitative polymerase chain reaction (RT-PCR)

Total RNA was extracted with TRIzol® reagent (Invitrogen). Primers were synthesized by Takara Biomedicals. The β-actin is used as internal control. The sequences for Akt1, p53 and PTEN, as well as β-actin were listed in Table 1. The PCR system (50 μl) was performed in 25 μl of SYBR^R^ Green Realtime PCR Master Mix (2×, obtained from Toyobo), 2 μl of each primer, 2 μl of DNA template and distilled water (15 μl). The PCR procedures were under the following conditions: an initial denaturation step (95°C for 15 s) and denaturation (95°C for 5 s), annealing step (72°C for 10 s) in a total of 40 cycles. Finally, the PCR results were analysed with the 7700 Sequence Detection System (Applied Biosystems). β-Actin was used as an internal reference, the average value of each sample was analysed with three parallel tubes. The expression of mRNA within groups was detected by using relative quantitative method. The relative value of mRNA was expressed using 2^−ΔΔCT^ [ΔΔCT=(CTmRNA - CTβ-actin) experimental group–(CTmRNA—CTβ-actin) control group]. And the triple times repetition test of the same sample indicated that the FQ-PCR was reproducible.

**Table 1:** The sequences for *Akt1*, *p53* and *PTEN*, as well as *β-actin* U, upstream; D, downstream.

		Primer sequences
*Akt1*	U	5'-ATGAGCGACGTGGCTATTGTGAAG-3'
	D	5'-GAGGCCGTCAGCCACAGTCTGGATG-3'
*p53*	U	5'-TTGGATCCATGTTTTGCCAACTGGCC-3'
	D	5'-TTGAATTCAGGCTCCCCTTTCTTGCG-3'
*PTEN*	U	5'-GAGGGAATAAACACCATG-3'
	D	5'-AGGGGTAGTGAGTGACACAGTA-3'
*β-actin*	U	5'-CAGAGCCTCGCCTTTGCC-3'
	D	5'-GTCGCCCACATAGGAATC-3'

### Western blot detection

Total protein was extracted using a BCA Protein Assay Kit (Wuhan Boster Biological Engineering). After the adding of 300 ul tissue lysates (Beijing Biosynthesis Biotechnology) for 15 min, samples were separated by SDS/PAGE system, and transferred to PVDF membrane with 5% blocking buffer at 4°C for 3 h. Adding primary antibodies of goat anti-human Akt1 antibody, rabbit anti-human p53 and PTEN antibodies (1:1000; Abcam), the member was incubated overnight at 4°C, washing three times for 15 min with PBS buffer. Subsequently, horseradish peroxidase-conjugated goat anti-rabbit IgG (secondary antibody, diluted with 3% BSA at a ratio of 1:1000) was added, incubating at 37°C for 1 h and washing three times for 15 min with PBS. All the primary and secondary antibodies were purchased from Santa Cruz. Thereafter, samples were coloured with DAB and were then scanned. All the experimental procedures were repeated for three times.

### *In vivo* establishment of mouse model

A total of 40 mice were randomly selected, and then the collected OVCAR-3 cells were subcutaneous inoculated in the right anterior axillary of each mouse (1×10^7^ cells each). After 5 days growth, subcutaneous tumour nodules were palpable in each mouse, and the average diameter of the tumour nodule was approximately 5 mm after 7 days inoculation.

Thereafter, 7 days after the inoculation of OVCAR-3 cells, the OV-H1 fusion cell, H1 hESCs and OVCAR-3 ovarian cancer were injected into 10 mice (100 μl each) respectively; and the same volume of PBS were injected in the remaining mice as the control group. To observe the tumour growth and to calculate the volume of the tumour, the two longest diameter of the tumour were calculated combined with the formula: *V*=1/6*πR*_1_^2^*R*^2^.

### TUNEL apoptotic cell detection

The mice were killed at 7 days, 28 days and 35 days after inoculation with the parental and the hybrid cells. The tumour tissues were fixed with 10% formalin and embedded in paraffin. The TUNEL assay for apoptosis detection was made into 6 μl thick slices according to the protocols of the manufacture.

### Statistical analysis

The data were analysed by SPSS 20.0 software, the measurement data were analysed by *t* test, which were presented by means ± S.D., the enumeration data were analysed by chi-squared test, *P*< 0.05 was considered as statistically significant.

## RESULTS

### Lower trend of fusion cell growth observed in OV-H1 cells

**Figure 1:**
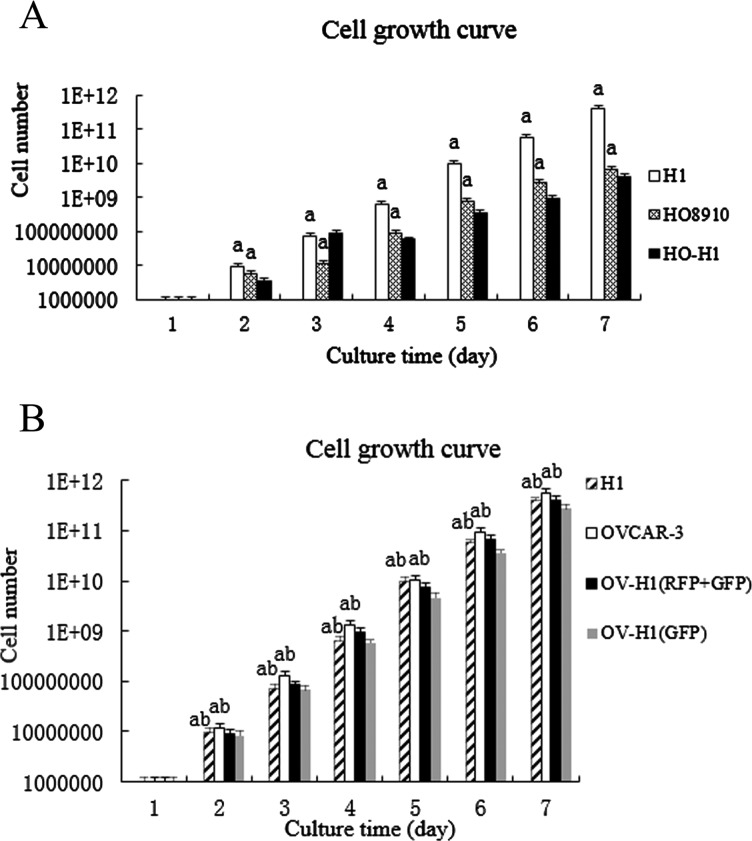
Growth curves of hybrid cells after fusion (**A**) The growth curve of hybrid cells after fusion of H1 and HO8910 cells. (**B**) The growth curve of hybrid cells after fusion of H1 and OVCAR-3 cells. Note: a, compared with the other cells, *P*< 0.05. ^b^, compared with the hESCs and OVCRA-3 ovarian cancer cells, *P*>0.05.

The growth of OV-H1 (RFP+GFP) hybrid cells with double fluorescence expressions were obviously slower than that of hESCs and OVCRA-3 ovarian cancer cells, with no statistically difference. The growth of OV-H1 (GFP) hybrid cells with green fluorescence expression was slower than that of the other three cells (*P*< 0.05); the growth of HO-H1 hybrid cells was slower than that of the two parental cells (*P*< 0.05) (Figure 1).

### *p53* and *PTEN* gene expressions were greatly suppressed in fusion cells than in parental cells

*P53* and *PTEN* gene expressions in OV-H1 (RFP+GFP) cells were obviously lower than those in the two parental cells, which were statistically significant (both *P*< 0.05). *P53* and *PTEN* gene expressions in OV-H1 (GFP) cells were obviously lower than those in the parental cells; however, there was no difference from H1. P53 expression in HO-H1 cells was higher than those in the two parental cells, which was significantly different among the three types of cells. PTEN expression in HO-H1 cells was higher than that in the H1 cells and lower than that in the OVCRA-1 cells, which was significantly different among the three types of cells (Table 2).

**Table 2:** Comparison of *p53* and *PTEN* gene expressions in fusion cells and parent cells

Group	Number (*n*)	*p53* expression positive (*n*, %)	*χ*^2^	*P*	*PTEN* expression positive (*n*, %)	*χ*^2^	*P*
HO-H1 fusion cells	30	5 (16.67%)			6 (20.00%)		
H1 cells	30	10 (33.33%)			11 (36.67%)		
OVCRA-3 ovarian cancer cells	30	24 (80.00%)	5.305	<0.05	25 (83.33%)	6.398	<0.05

### Apoptosis signal of the OV-H1 cells was higher than that of the H1 and OVCAR-3 cells

**Figure 2:**
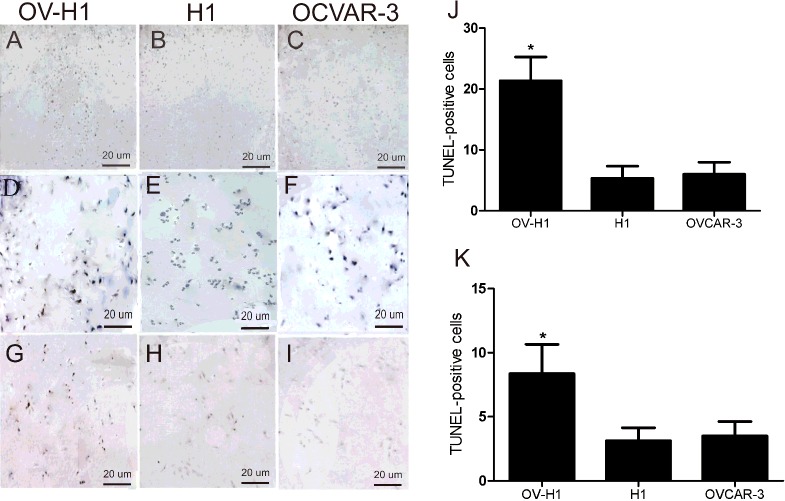
TUNEL apoptotic cell detection (**A**–**I**) The TUNEL *in situ* cell apoptosis detection results of the apoptosis signal of the OV-H1 hybrid cells, the of hESCs and OVCAR-3 ovarian cancer cells. (**J**–**K**) The number of positive cells.

TUNEL *in situ* cell apoptosis detection results showed that the apoptosis signal of the OV-H1 hybrid cells was significantly higher than that of the of H1 and OVCAR-3 ovarian cancer cells (Figures 2A–2C). In the experimental group near hESCs, there were more TUNEL-positive signals in the OV-H1 hybrid cells and the rest showed few scattered (Figures 2D–2F). The number of positive cells was counted by eight visual fields (100×, Figure 2J). Further, caspase-9 apoptosis test results also showed that the apoptotic signal intensity in the experimental group was significantly stronger than that in the control group (Figures 2G–2I). The number of positive cells was as shown in Figure 2(K) counted by six visual fields (100×).

### Akt1, p53 and PTEN in OV-H1 hybrid cells were weaker by quantitative RT-PCR

**Figure 3:**
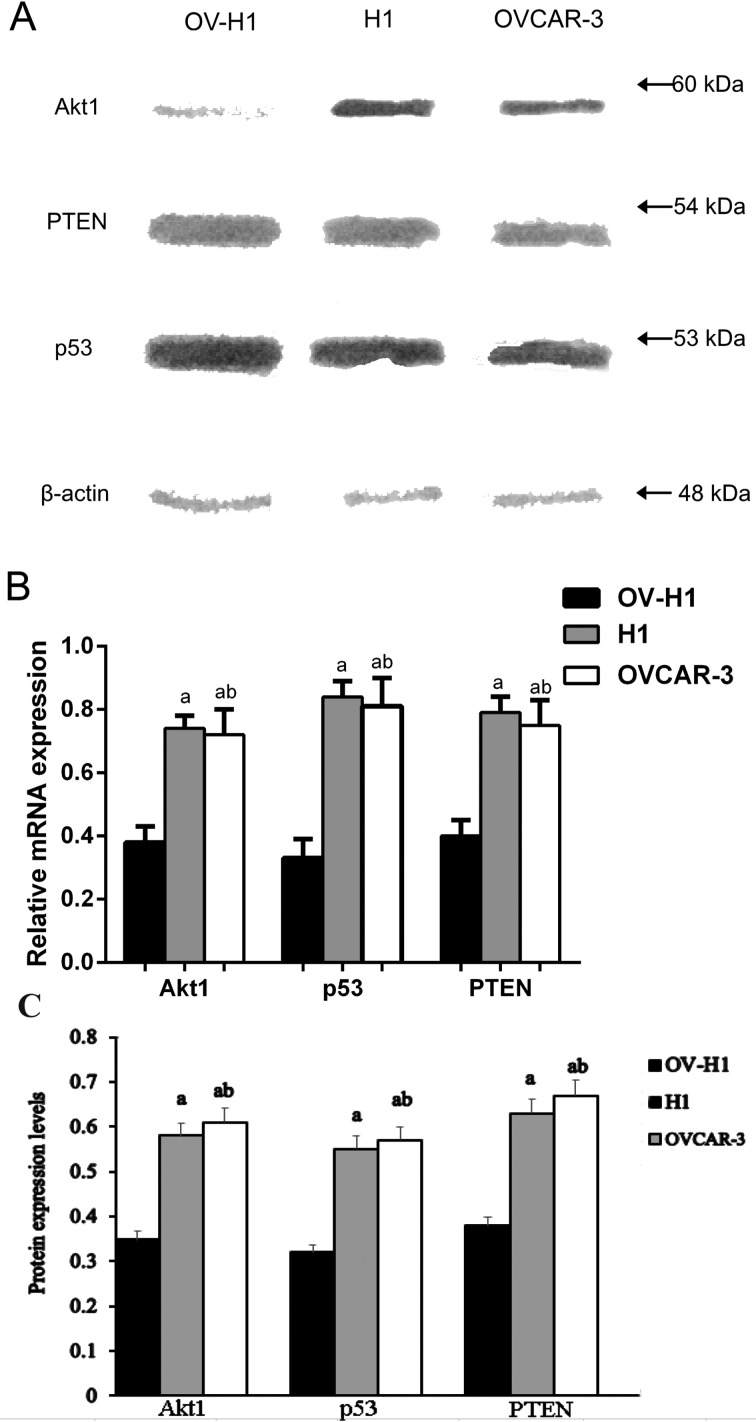
Quantitative RT-PCR and western blot results (**A**) Gel electrophoresis map showing the electrophoresis bands of Akt1, p53 and PTEN following PCR procedures. (**B**) The relative mRNA expressions levels of Akt1, p53 and PTEN were detected by real-time fluorescence quantitative polymerase chain reaction. (**C**) The protein expressions of Akt1, p53 and PTEN were detected by Western blot. Note: a, compared with the OV-H1 hybrid cells, *P*< 0.05; ^b^, compared with the values between hESCs and OVCAR-3 ovarian cancer cells, *P*>0.05.

The mRNA expressions of Akt1, p53 and PTEN in the OV-H1 hybrid cells were much weaker than those of the hESCs and OVCRA-3 ovarian cancer cells, whereas no significant statistical difference was found between the hESCs and OVCRA-3 ovarian cancer cells (Figures 3A–2B).

### Akt1, p53 and PTEN protein expression detected by western blot

With β-actin as the reference, the western blot results indicated that the protein expression of Akt1 in the OV-H1 hybrid cells was significantly weaker than that in the hESCs and OVCAR-3 ovarian cancer cells. In addition, the protein expression of p53 and PTEN were both decreased than that in the hESCs and OVCAR-3 ovarian cancer cells. Each test was repeated three times. Results were shown in Figure 3(C).

### *In vivo* results of tumours growth were inhibited after inoculation of OV-H1

**Figure 4:**
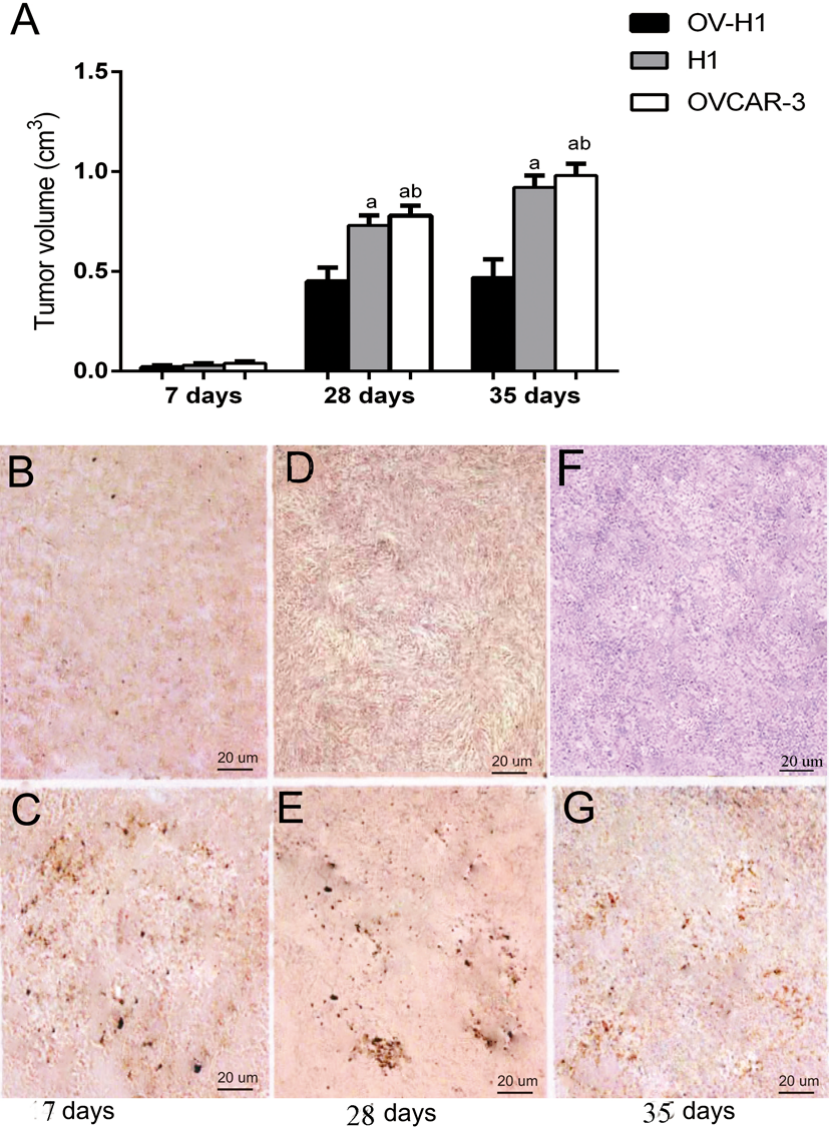
*In vivo* results of tumors growth and cells apoptosis (**A**) *In vivo* results of tumours growth between groups at different time point. Note: a, compared with the OV-H1 hybrid cells, *P*< 0.05; ^b^, compared with the values between hESCs and OVCAR-3 ovarian cancer cells, *P*>0.05. (**B**–**G**) *In vivo* results of TUNEL apoptotic cell detection at 7 days, 28 days and 35 days. (**B**, **D** and **F**) The experimental group; (**C**, **E** and **G**) the control group.

Compared with 7 days, 28 days and 35 days after inoculation of OV-H1 hybrid cells, tumour volumes in the experimental groups was significantly smaller than those of the control group at 28 days and 35 days, the tumour volume curve was shown in Figure 4(A). Besides, within the experimental groups, tumour volumes in mice inoculated with OV-H1 hybrid cells was much smaller than those inoculated with hESCs and OVCAR-3 ovarian cancer cells. In addition, when compared with the tumour volume 7 days after inoculation, there were obvious difference when compared with that 28 days and 35 days after inoculation, showing significantly increase (both *P*< 0.05).

### *In vivo* results of TUNEL apoptotic cell detection

In 7 days, 28 days and 35 days, the mice were killed, and the tumour tissue sections were used for TUNEL apoptosis detection. The results showed that in different time period, the apoptotic signal of the experimental groups was stronger than that of the control group, much stronger apoptotic signal was found in OV-H1 hybrid cells inoculated mouse. Meanwhile, the distribution of apoptotic signal in tumour tissues gradually dispersed overtime, as shown in Figures 4(B)–4(G).

## DISCUSSION

By cell fusion, previous researchers have found many anti-tumour genes and acknowledged the function and effect of tumour inhibition genes [28–30]. Hybrid cell of cancer cell and somatic cell is applied to produce monoclonal antibody [31]. The tumour cell fusion *in vivo* have verified that the hybrid cell of cancer cell and somatic cell chimeras is a kind of cell with higher malignancy, which is manifested as the increase in migration, drug resistance, proliferation rate, and decrease in apoptotic rate [32–34]. For some cancer cells, the spontaneous fusion *in vivo* has higher proliferation ability than the cell fusion induced by PEG, and even the apoptotic rate is lower [35–37]. After cancer cells migrate to other tissues, they can fuse with the cells in the tissue to generate immunogenicity [38]. After fusion, adult somatic cells can show the characteristics of stem cells, that is to say, the cells have potential of self-renewability and differentiating to many types of cells [39–41]. Cell fusion has become the important method in multipotential stem cell research and in-depth exploration of somatic cell reprogramming mechanism.

In the present study, after the fusion of hESCs and OVCAR-3 cells, we first observed the role of hESCs in the growth of OVCAR-3 cells *in vitro* and *in vivo*. HE staining and cells count results showed that the growth of OVCAR-3 cells was significantly inhibited after the con-fusion with hESCs and thereby OVCAR-3 cells apoptosis was detected. Following, to confirm the existence and effect of cells apoptosis, the expression of caspase-9 was also investigated. The apoptotic signal intensity of caspase-9 in the experimental group was significantly stronger than that in the control group. As the promoter of the apoptotic pathway and the Caspases family, caspase-9 plays an important role in the development of tumours, inactivated caspase-9 has been detected in several human tissues such as in heart, testis and ovary. Previous evidence also showed that ovarian cancer cell lines, including the OVCAR-3 cells were detected without the expression of caspase-9, which was in line with the present results. Besides, the positive signal of caspase-9 after fusion was significantly stronger than those without fusion, which was in accordance with the results of TUNEL detection, suggesting that hESCs inhibiting the growth of OVCAR-3 cells was possibly achieved by the activation of the Caspases family and induction of OVCAR-3 cells apoptosis.

In the past decade, people have found that embryo derived stem cells including MSC, embryonic carcinoma cells and embryonic germ cells which can reprogram adult somatic cells to have MSC potentials by changing gene expression model, development status and epigenetics regulation of adult somatic cells [42–44]. Stem cell is a kind of cell which has self-renewal and differentiation function [45,46]. Under the specific condition, stem cell can be induced to express some specific genes, and differentiated cells can substitute injured cells or correct some diseases, thus stem cell can be used for cell therapy [47–49]. The self-renewal and multi-directional differentiation potential can maintain the normal balance by regulation of some signalling pathways. With respect to the present investigation on apoptosis which was regulated by multiple factors, the signalling pathway is one of the critical target. As the upstream signal pathway of caspase-9, AKT signalling pathway belongs to the phosphatidylinositol 3-kinase (PI3K) family that is responsible for the regulation of cell functions, such as proliferation, differentiation, apoptosis and glucose transport [50,51]. PI3K/AKT signalling pathway has been reported to be disordered in a wide range of human tumour spectrum [52–55]. Western blot and quantitative RT-PCR results showed that both the protein expression and mRNA expression of Akt1 following fusion was significantly weaker than that in the cells without fusion, which in turn highlighted that hESCs inhibited the mRNA expression of AKT and the phosphorylation of AKT protein, and eventually contribute to the activation of caspase-9 and the apoptosis of OVCAR-3 cells.

Another important result of the present study was that fusion of hESCs and OVCAR-3 ovarian cancer cells could have an influence in OVCAR-3 cells growth via the exerting of p53 and PTEN effects. P53 serves as a tumour suppressor functioning significantly in inhibiting tumour angiogenesis [56]. Importantly, P53 is closely related to the survival and differentiation of stem cells. hESCs are prone to spontaneous apoptosis or differentiation, not easy to save and culture, and the expression of p53 is suggested to reduce the apoptosis, differentiation and differentiation rate of hESC helpfully [57]. In addition, PTEN serves as an antagonist of the PI3K/AKT pathway, and this pathway is an oncogenic pathway because of its role in cell cycle control and cell proliferation [58]; additionally, loss of PTEN expression is significantly related to the activation of the PI3K/AKT pathway [59]. Hence, PTEN negatively modulates the PI3K/AKT pathway, and thereby may have a crucial effect on the control of cell cycle and cell survival [60]. Consequently, loss of PTEN normal growth regulation, inhibiting inducers of apoptosis and promoting cell survival by phosphorylation may thereby favoured tumour formation. In the present study, p53 and PTEN expression were decreased compared with parental cells after fusion of ovarian cancer cells and hESCs, which in turn confirmed the successful establishment of new growth model. Importantly, epigenetic changes that were associated with genes expressions including p53, PTEN, Akt1 were somewhat confirmed in the present study. The achievement of epigenetic changes was mainly depend on histone acetylation and DNA methylation so as to achieve the goal of preventing and treating tumours [61], to be specific, histone acetylation can activate the transcription of specific genes which is critical for cells differentiation and proliferation; on the other hand, DNA methylation is mainly manifested by the decrease in the overall methylation level of the genome and the abnormal increase in the degree of methylation in the local CpG islands. The latter can lead to the expression of some tumour suppressor genes and participate in the occurrence and development of tumour. In view of the above, either histone acetylation or DNA methylation, or both might be involved in the process of epigenetic changes that were associated with p53, PTEN, Akt1 expressions. Due to the restriction of experiment time and costs, there was no further in-depth research of histone acetylation or DNA methylation in the present study. However and beyond a doubt, we quite agree and support the idea that tumour often correlated with the in-activation of multiple anti-oncogene, previous single gene therapy could not inhibit the growth of tumours successfully, methylation or deacetylate enzyme inhibitors targeting the whole genome could help to the recovering of multiple anti-oncogene expressions, therefore decreased gene mutation and increased genomic stability rates, so as to provide new targets for drug development of human tumours [62]. Such important and interesting topic is definitely planned to be studied in our following research.

It is reported that the random integration of exogenous gene may affect the expression model of whole-genome and gene stability, influencing cell function or increase carcinogenesis risk [63]. By identifying the cytobiological features of transgenic cells, it is found that the integration of exogenous gene mediated by lentivirus does not affect the biological characteristics of stem cell such as self-renewal and multi-directional differentiation *in vivo* and *in vitro*. In the present study, we prepared pFinal/PGK-puro-EF1α-tdTomato and pFinal/PGK-BSD- EF1α-hrGFP carrier vectors using gateway technology. By the packaging and concentration of lentivirus, we obtained virus solution with high titre and biological safety. After further transduction and screening, we successfully established four transgenic cell lines carrying target genes. This indicates that gateway technology combined with lentivirus transduction vector provides an effective and convenient transduction system for human embryonic stem cells and tumour cells. The comparison of growing speed of hybrid cells and parent cells after fusion showed that the growth of hybrid cells derived from ovarian cancer cells was slower than those of the two parental cells. In the previous research, the cancer cell fusion *in vitro* showed that the hybrid cells of cancer cells and adult somatic cells had higher malignancy [64], manifesting as the increase in migration, drug resistance, proliferation rate, and decrease in apoptotic rate.

In conclusion, p53 and PTEN expression were decreased compared with parental cells after fusion of ovarian cancer cells and hESCs, the growth status was changed, the proliferation rate was decreased and a new growth model was established. Collectively, hESCs can inhibit the growth of OVCAR-3 cells *in vitro* by suppressing p53 and PTEN expression to inhibit the growth of tumour achieved by inducing apoptosis of OVCAR-3 cells. Thus, the change of epigenetics after fusion of ovarian cancer cells and hESCs may become a novel direction for treatment of ovarian cancer.

## Abbreviations

DAB: 3,3′-diaminobenzidine tetrahydrochloride
DMEM: Dulbecco's Modified Eagle's Medium
hESC: human embryonic stem cell
MEF: mouse embryonic fibroblast
PI3K: phosphatidylinositol 3-kinase
TdT: terminal dexynucleotidyl transferase
